# Prevalence and cervical organism burden among Louisiana women with *Trichomonas vaginalis* infections

**DOI:** 10.1371/journal.pone.0217041

**Published:** 2019-06-20

**Authors:** Meredith K. Shaw, Harry S. Porterfield, Sue Favaloro, Patricia M. Dehon, Barbara Van Der Pol, Alison J. Quayle, Chris L. McGowin

**Affiliations:** 1 Department of Microbiology, Immunology, and Parasitology, Louisiana State University Health Sciences Center, New Orleans, LA, United States of America; 2 Department of Pathology, Louisiana State University Health Sciences Center, New Orleans, LA, United States of America; 3 Molecular Pathology Laboratory, University Medical Center, New Orleans, LA, United States of America; 4 Division of Infectious Diseases, University of Alabama Birmingham School of Medicine, Birmingham, AL, United States of America; 5 Department of Internal Medicine, Section of Infectious Diseases, Louisiana State University Health Sciences Center, New Orleans, LA, United States of America; University of Texas Health Science Center at San Antonio, UNITED STATES

## Abstract

*Trichomonas vaginalis* is the most common curable sexually transmitted infection (STI) worldwide. Although predominately asymptomatic, the disease spectrum of trichomoniasis in women is characterized primarily by signs and symptoms of vaginitis, including purulent discharge and localized vulvar pruritus and erythema. Several FDA-cleared nucleic acid amplification tests (NAATs) are available for the diagnosis of *T*. *vaginalis* infections, but laboratory developed tests (LDTs) are widely utilized and cost-effective solutions in both the research and clinical diagnostic settings. LDT diagnosis of *T*. *vaginalis* is particularly appealing since it can be performed using remnant specimens collected for other STI testing. Using a LDT implemented as part of this study, *T*. *vaginalis* was detected in 7% of participating Louisiana women (14/199). The mean *T*. *vaginalis* organism burden was 1.0x10^6^ ± 4.5x10^5^ organisms per mL of ThinPrep PreservCyt. Using DNA eluates obtained after HPV testing on the cobas 4800 system, the *T*. *vaginalis* LDT was characterized by excellent intra- and interassay reproducibility (coefficient of variation values all <3.5%). Compared with two commercially available NAATs from TIB MOLBIOL, the sensitivity and specificity of the LDT was 92.9 and 99.5%, respectively. Collectively, this study details the diagnostic and quantitative utility of a LDT for *T*. *vaginalis*. When applied in the clinical research setting, we confirmed the high prevalence of *T*. *vaginalis*, but also observed extraordinarily high organism burdens in the cervix. These findings highlight the unique host-pathogen relationship of *T*. *vaginalis* with lower reproductive tract tissues, and substantiate the need for continued investigation of this highly prevalent STI.

## Introduction

*Trichomonas vaginalis* is the most common curable sexually transmitted infection (STI) worldwide [1]. In the USA, *T*. *vaginalis* is more common than *Chlamydia trachomatis* and *Neisseria gonorrhoeae* in nationally representative studies [2, 3], however no programmatic recommendations currently exist for routine screening in the general population [4]. Unfortunately, timely identification of trichomoniasis is further complicated by the fact that up to 85% of infections in women are asymptomatic [5], despite being associated with inflammatory lower urogenital tract syndromes including vaginitis and cervicitis. Ascending infections have also been associated with pelvic inflammatory disease (PID) [6, 7] and several adverse pregnancy outcomes including preterm birth [8, 9]. Clinically, trichomoniasis is most commonly associated with signs and symptoms of vaginitis including purulent vaginal discharge, and localized vulvar pruritus and erythema. Although oral metronidazole or tinidazole are highly effective therapies [10], timely diagnosis of *T*. *vaginalis* remains sub-par as we most commonly rely upon insensitive in-clinic techniques, and direct our diagnostic testing towards patients with overt signs of vaginitis.

Nucleic acid amplification tests (NAATs) are the gold standard for *T*. *vaginalis* diagnosis, although microscopy of vaginal wet mount specimens is still the most commonly employed method today [11, 12]. The sensitivity of diagnostic wet mount microscopy is only 40–60% compared to contemporary NAATs [13], several of which are FDA-approved for diagnostic use in the USA [12]. Culture-based tests have similar sensitivity to NAATs, but are rarely used outside of the research setting since the turn-around times are long relative to both wet mount and NAATs. Although few exist, point of care (POC) tests have clear benefits over send-out testing as they reduce empiric therapy for *T*. *vaginalis* and other STIs. Currently, the available POC solutions cannot simultaneously test for other infectious etiologies of vaginal discharge (e.g. chlamydia and gonorrhea) and therefore parallel POC and/or laboratory testing is still warranted for a complete evaluation. Medium to high-throughput NAATs, such as those typically performed in hospital and reference laboratories, are cost-effective for clinic-wide or local population-based screening of *T*. *vaginalis* in asymptomatic populations. These tests are particularly applicable when an immediate turn-around time is not imperative, and can also be used for chlamydia and gonorrhea screening from the same specimen. Laboratory developed tests (LDTs) are widely utilized for infectious disease testing in both the clinical diagnostic and research settings, and can be easily scaled within the laboratory based on the volume of test requests. Using a newly-developed LDT for *T*. *vaginalis* detection in liquid-based cytology specimens, we assessed prevalence and cervical organism burden in a cohort of Louisiana women. As part of Papanicolaou-based cervical cancer screening [14, 15], liquid cytology specimens are collected at regular intervals from 21–65 years of age and facilitate STI screening in older age groups not typically targeted for annual STI screening.

## Materials and methods

### Assay design and target specificity

The primers and TaqMan probe were designed manually to target a 105 bp region of the *serine hydroxymethyltransferase* gene (TVAG_109540; See Table 1). Analytical specificity of the primer and probe design was initially assessed *in silico* via BLASTn query of the NCBI nucleotide (nt) database, and then confirmed by testing a panel of urogenital pathogens and non-pathogens. The panel included DNA (1-5x10^4^ genomic equivalents per reaction) purified from laboratory cultures of *Mycoplasma genitalium* strain G37 (ATCC 33530), *Mycoplasma pneumoniae* (ATCC 15531), *Chlamydia trachomatis* serovar D, *Trichomonas tenax* (Muller) Dobell (ATCC 30207), Herpes Simplex Virus type II, *Mycoplasma hominis* (ATCC 23114), *Mycoplasma pirum* (ATCC 25960), *Neisseria gonorrhoeae* (ATCC 700825), *Mycoplasma fermentans* strain S38, *Ureaplasma urealyticum* (ATCC 27814), and one clinical isolate each of *Gardnerella vaginalis*, *Candida albicans*, *Lactobacillus jensenii*, and *Ureaplasma parvum*. General cross-reactivity to normal cervicovaginal microflora was assessed by testing 25 PreservCyt specimens (Hologic, Inc., Bedford, MA) that previously tested negative with the Hologic Aptima *T*. *vaginalis* test (Hologic, Inc.) on the Panther system. The capacity to detect different clinical isolates of *T*. *vaginalis* was assessed using DNA purified from 25 otherwise-discarded *T*. *vaginalis*-positive InPouch TV cultures (BioMed Diagnostics, White City, OR) of vaginal specimens obtained at the CrescentCare Sexual Health Clinic in New Orleans, LA.

**Table 1:** Primer and TaqMan probe sequences for the *T*. *vaginalis* LDT.

	Target Region*	Oligonucleotide Sequence	Melting Temperature
Forward Primer (TV109540_1F)	1206–1184	5’-CCATCAAGAGCATGCTTAGCTGC-3’	58.6°C
Reverse Primer (TV109540_1R)	1101–1126	5’-GTTCATCAACGTATTTGGTGCCTCCA-3’	59.4°C
TaqMan Probe (TVAG_109540-TP)^†^	1160–1132	5’-AGTATGCGGAAGGATATCCAGGTGCTCGC-3’	64.6°C

* Numbering relative to the 1,356 bp Serine hydroxymethyltransferase gene TVAG_109540 from Genomic Reference Sequence NW_001581720.1.

^†^ The TaqMan probe is modified with a 5’ 6-FAM fluorophore, an internal ZEN quencher, and a 3’ Black Hole Quencher 1.

### Population characteristics and specimen collection

LSUHSC University Medical Center Molecular Pathology Laboratory analyzed de-identified ThinPrep PreservCyt samples following an approved protocol by the LSUHSC Institutional Review Board (IRB). Informed consent was not required to participate in this study. Subjects contributing specimens for this study (n = 200) attended one of five LSU-affiliated hospitals and women’s clinics in Louisiana in 2014 (the percentage of specimens contributed from each site are noted in parentheses): Earl K. Long Hospital, Baton Rouge, LA (5.9%); Leonard J. Chabert Medical Center, Houma, LA (18%); Lallie Kemp Regional Medical Center, Independence, LA (5.5%); the Interim LSU Hospital, New Orleans, LA (33.3%); University Medical Center, Lafayette, LA (6.8%); W.O. Moss Regional Medical Center, Lake Charles, LA (3.4%); and LSU Bogalusa Medical Center, Bogalusa, LA (27.1%). Specimens were collected during pelvic exam using either a cervical broom or the cytobrush/spatula combination. All specimens were collected initially for cytology-based cervical cancer screening and processed on the ThinPrep 2000 automated slide preparation system (Hologic, Inc.). Following cytological processing, specimens were transferred to the Molecular Pathology lab and, if ordered, processed for HPV testing on the cobas 4800 system (Software Version 2.1). Subject age was abstracted and then the samples were de-identified for *T*. *vaginalis* LDT testing; all LDT results were obtained within 45 days of initial specimen collection.

### DNA purification

With the goal of validating both a high- and low-throughput extraction method, this study assessed LDT performance characteristics using spin columns and also residual DNA eluates from the automated x 480 extraction component of the cobas 4800 system. DNA eluates remaining after HPV testing were obtained directly from the cobas 4800 system, and were then frozen at -20°C until PCR was performed. To assess stability, residual DNA eluates were spiked with 1.5x10^5^ copies of genomic *T*. *vaginalis* DNA and then subjected to once-weekly freeze-thaw cycles for 21 days, with and without magnetic glass particles, followed by LDT testing after each freeze-thaw cycle.

For the comparison of DNA extraction methods, a panel of LDT-positive/negative specimens (n = 20 each; ascertained using residual x 480 eluates) was extracted using spin columns, the method of which is described below. LDT results were then compared directly for Kappa calculations. For spin column-based preparations, 400 μL of the ThinPrep specimen was transferred to a 1.5 mL microfuge tube and centrifuged at 2000 x g for 5 minutes at room temperature. To enable direct comparison of the extraction methods, the 400 μL specimen volume was chosen to match that processed by the cobas 4800 for HPV testing. Supernatants were carefully removed leaving a volume of approximately 100 μL; this ensured the pellet was not disturbed and maximized specimen input for DNA extraction. Lysis buffer (180 μL, buffer ATL; Qiagen, Inc, Valencia, CA) was then added and processed using the DNeasy Blood and Tissue Kit per the manufacturer’s instructions. Specimens were eluted in 200 μL of Qiagen buffer AE and stored at -20°C until PCR was performed. DNA quantity and purity were measured by spectrophotometry (NanoDrop 2000; Thermo Fisher Scientific, Waltham, MA). For *T*. *vaginalis* testing using the TIB MOLBIOL kits (Berlin, Germany), we adhered to the package insert and utilized spin column-purified DNA; no comparative assessment of extraction methods for subsequent *T*. *vaginalis* detection was performed.

### Nucleic acid amplification tests (NAATs)

The TaqMan-based quantitative PCR procedure developed in our laboratory has been described previously for *T*. *vaginalis* detection [16], but modified as follows using the LightCycler FastStart DNA Master Hybridization Probes M^GRADE^ Reagents (Roche Diagnostics): 2.0 μL FastStart Enzyme/Reaction Buffer mix, 3.2 μL MgCl_2_ stock solution (25 mM), 6.8 μL water, 1.0 μL forward primer (5 μM stock), 1.0 μL reverse primer (5 μM stock), 1.0 μL TaqMan Probe (5 μM stock), and 5.0 μL of DNA template. Cycling parameters consisted of an initial denaturation step at 95°C for 5 min, followed by 40 cycles of 95°C for 10 sec, 53°C for 30 sec, and 72°C for 10 sec. In order to quantify *T*. *vaginalis* DNA loads in ThinPrep PreservCyt specimens, a standard curve (10-fold serial dilutions) of pre-quantified plasmids encoding the target amplicon was tested in parallel on each run; the standard curve was prepared immediately prior to each run.

In addition to our LDT, two *T*. *vaginalis* tests from TIB MOLBIOL (Berlin, Germany) were comparatively assessed in this study: (1) LightMix *Trichomonas vaginalis*; and (2) LightMix Modular Dx *Trichomonas vaginalis* Repeat DNA. Both TIB MOLBIOL tests were performed as detailed in the package insert using spin-column-purified DNA as described above. In a separate reaction, the human β-globin gene was targeted for a general marker of specimen adequacy as previously described [17] from both the spin-column purified DNA and residual cobas 4800 DNA eluates. The lower limit of detection (LOD) was ascertained by spiking serial dilutions of purified *T*. *vaginalis* organisms (NATtrol Molecular Control, Zeptometrix Corp., Buffalo, NY) into a *T*. *vaginalis*-negative specimen (confirmed to be negative by Aptima), and then performing replicate (n = 12) LDT testing. All LDT and TIB MOLBIOL testing was performed on the cobas z 480 analyzer using the UDF software (version 1.0.0.15). ThinPrep specimens with discrepant results among the NAATs were then tested using the FDA-approved Aptima *T*. *vaginalis* test on the Panther system.

### Statistical analyses

Significant differences in DNA concentration and purity between the x 480 and spin column preparations were assessed using the Student’s *t*-test. Crossing point (CP) values, analogous to crossing threshold (Ct), from LDT testing of pre-freeze specimens compared to those observed after once-weekly freeze thaw cycles were assessed by ANOVA, followed by Dunnett’s Multiple Comparison Test. Significant differences are indicated when p<0.05 for all tests. Sensitivity, specificity, positive predictive value (PPV), negative predictive value (NPV), and Kappa values among the LDT and TIB MOLBIOL test results were all determined using standard methods. The LOD was ascertained using Probit regression analysis.

## Results

### Testing workflow, analytical performance, and reproducibility

The LDT workflow utilizes either spin column-purified DNA or residual DNA eluates from cobas HPV testing. Purified DNA is used as template in PCR reactions assembled manually in the 96-well amplification/detection plate. The thermocycling protocol and data acquisition requires approximately sixty minutes to complete. Using the second derivative max analysis mode, generating results requires no user input or interpretation of amplification curves within the cobas UDF software. When the LDT is run as a quantitative test as described herein, which includes running a standard curve of pre-quantified plasmid dilutions in duplicate, the maximum number of patient results per run (96-well plate format) is 84 (5 plasmid dilutions run in duplicate, 1 no template control, and one positive control are included in each run). As a qualitative test, the maximum number of patient specimens per run is 94 (1 no-template control and 1 positive control are included in each run). BLASTn searches of the NCBI nucleotide database using the sequences of each primer and the TaqMan probe indicated homology only to *T*. *vaginalis*. With regard to analytical specificity, one organism, *T*. *tenax*, demonstrated cross-reactivity with the LDT yielding a positive *T*. *vaginalis* result; otherwise, no positive results were observed (see Materials and Methods for organisms used in specificity testing).

**Fig 1:**
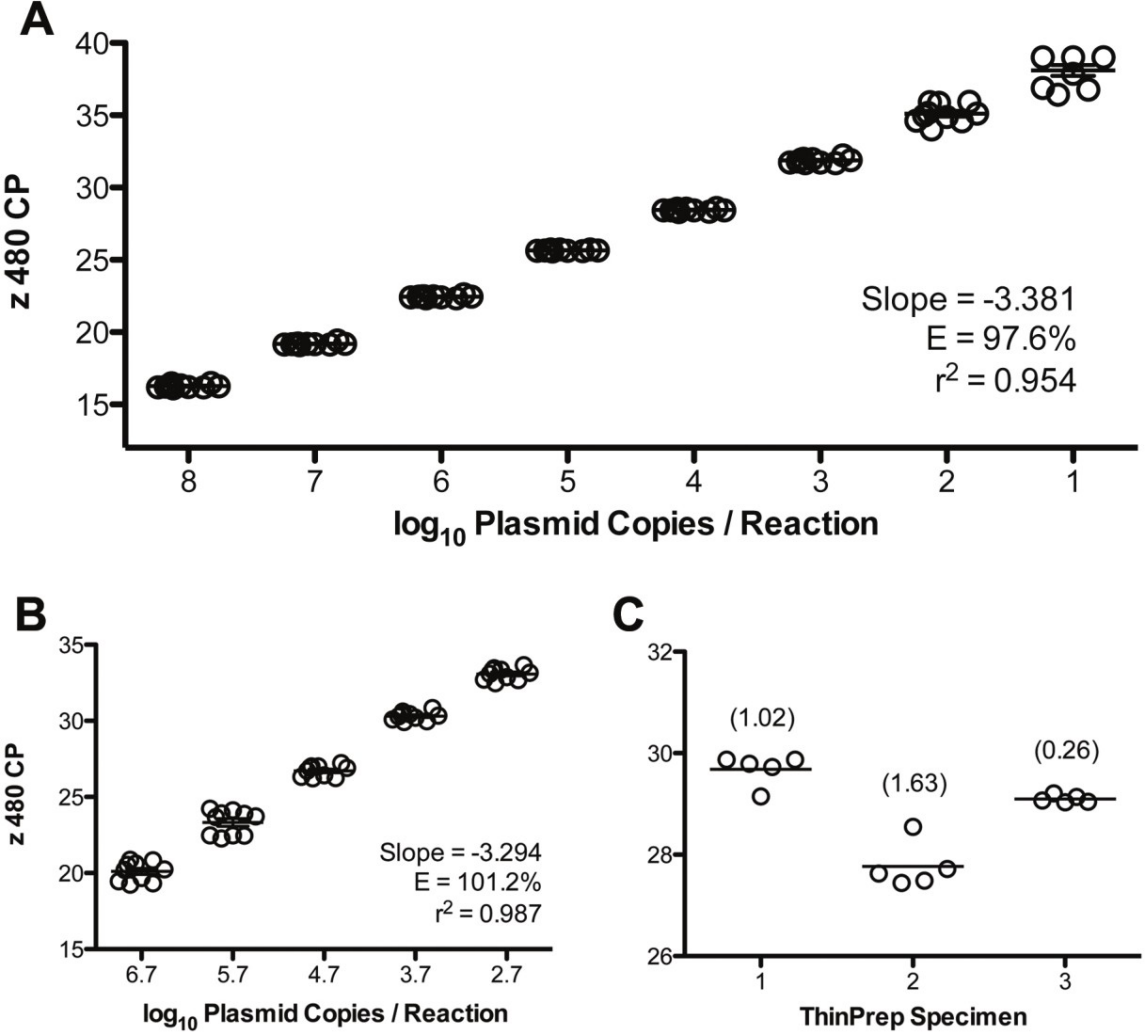
Reproducibility of the *T*. *vaginalis* LDT. (A) Intra-assay reproducibility and linearity of target detection was determined using ten replicates of pre-quantified, amplicon-encoded plasmids tested on a single run. (B) Inter-assay reproducibility was similarly assessed among ten LDT runs conducted over a five-week period. (C) Reproducibility of the entire LDT workflow was assessed using replicate DNA preparations (n = 5) of three *T*. *vaginalis*-positive ThinPrep PreservCyt specimens. Data are presented as crossing point (CP) values with % coefficient of variation among the replicates in parentheses. Slope, PCR efficiency (E), and r^2^ values are presented for the extended reproducibility data in panels A and B.

**Fig 2:**
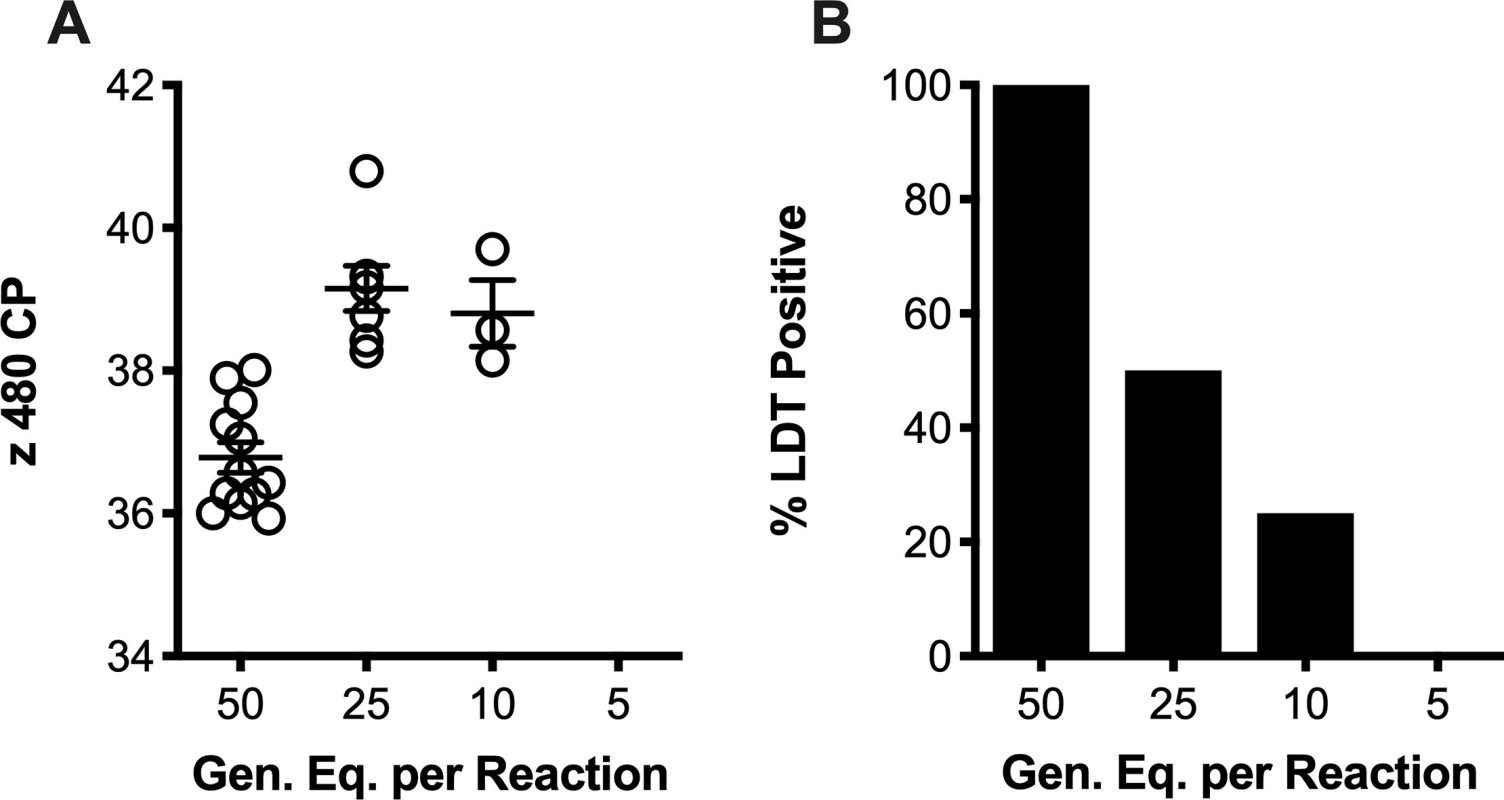
Empirical determination of the lower limit of detection (LOD). Serial dilutions of purified *T*. *vaginalis* organisms in ThinPrep PreservCyt specimens, corresponding to 50, 25, 10, and 5 genomic equivalents per reaction, were tested in replicate reactions (n = 12 each). Data are presented as (A) x 480 crossing point (CP) values, and (B) the percent of positive results observed from the replicate reactions for each of the serial dilutions. Probit regression analyses was used to accurately ascertain the LOD with 95% confidence. Data presented in this figure are representative of spin-column purified specimens; the Probit-derived LOD for spin column- and x 480-purified specimens is presented in the results section.

Using serial dilutions of pre-quantified plasmids encoding the *T*. *vaginalis* target amplicon (n = 10 replicates), intra-assay reproducibility was very good with coefficient of variation (CV) values less than 2.6 (Fig 1A). In the most diluted sample (10 copies per reaction), 9 of 10 reactions produced a positive result; all other replicate dilutions tested positive. Similarly, among ten LDT runs conducted over a five-week period, inter-assay reproducibility was very good as characterized by low CV values (<3.5%; Fig 1B). Reproducibility of the entire LDT workflow was assessed using replicate DNA preparations (n = 5) of three *T*. *vaginalis*-positive ThinPrep PreservCyt specimens. Replicate CP values for each of the three specimens was characterized by CV values of less than 1.7% (Fig 1C). Serial dilutions of *T*. *vaginalis* organisms in ThinPrep PreservCyt specimens, corresponding to 50, 25, 10, and 5 genomic equivalents per reaction, were detected in 100, 50, 25, and 0% of replicate reactions (n = 12 each; Fig 2), respectively. With 95% confidence, the LOD calculated by Probit regression analysis was 52.6 genomic equivalents per reaction, corresponding to 5.2x10^3^ and 3.9x10^3^ organisms per mL processed by spin-columns and the x 480, respectively.

### DNA extraction and eluate stability

**Fig 3:**
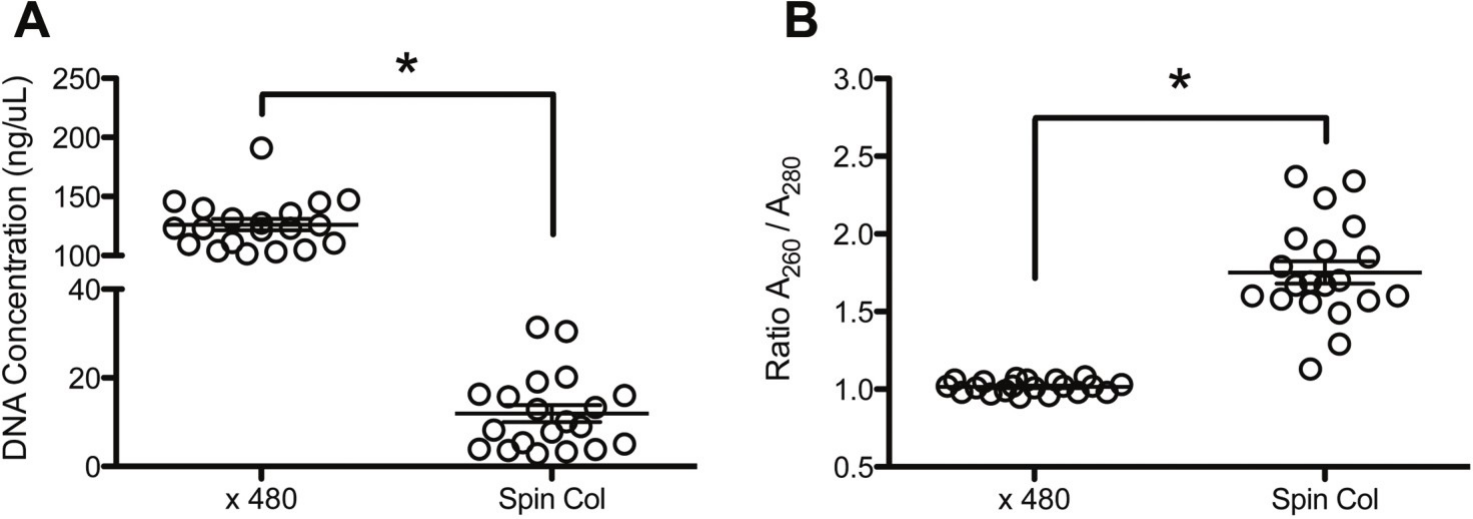
Comparison of DNA eluates from the cobas x 480 and spin columns. (A) Total DNA concentrations measured from x 480- and spin column-purified DNA eluates by NanoDrop spectrophotometry (absorbance at 260 nm; A_260_). (B) Crude DNA purity from the eluates was similarly assessed and expressed as a ratio of A_260_/A_280_. Asterisks denote significant differences in DNA concentration and purity between the extraction methods as determined by the Student’s *t*-test (p<0.05).

**Fig 4:**
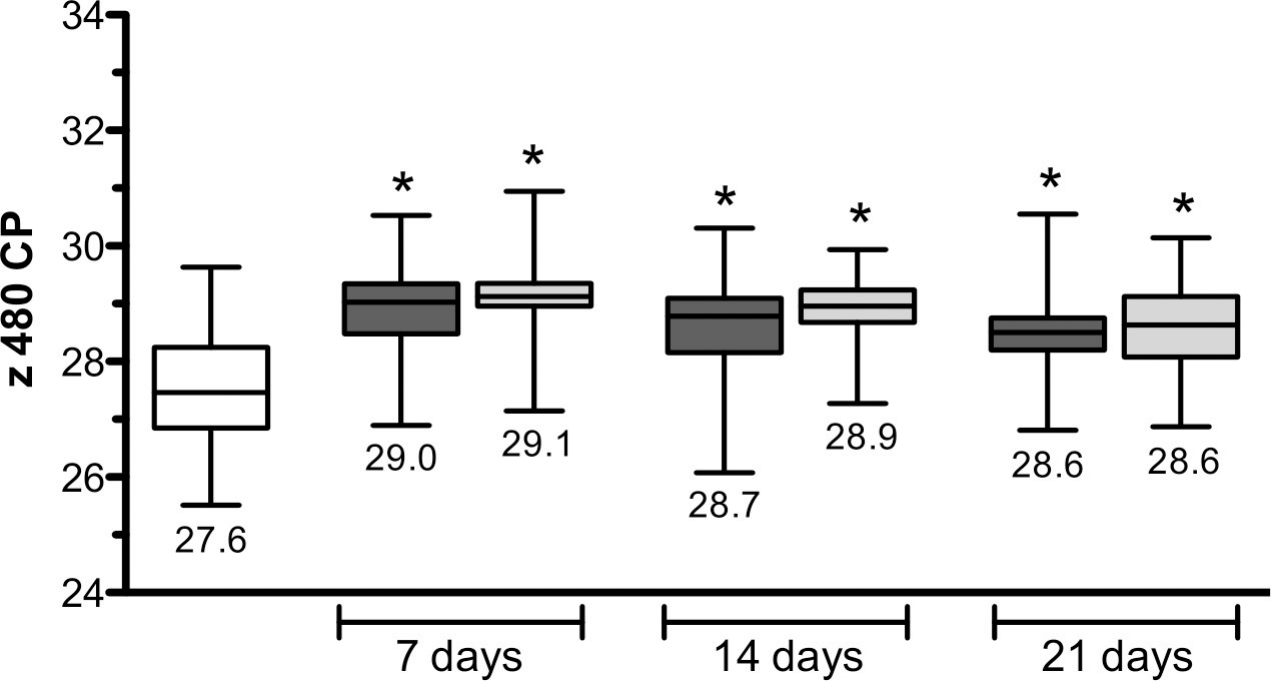
Freeze/Thaw stability assessment of x 480 DNA eluates. x 480 DNA eluates (n = 46 replicates) were spiked with 1.5x10^5^ copies of genomic DNA purified from a laboratory culture of *T*. *vaginalis*. Once-weekly freeze-thaw cycles for 21 days, without and with magnetic glass particles (dark grey and light grey fill, respectively), was compared to crossing point (CP) values obtained immediately after purification (pre-freeze; white fill). Data are expressed as CP values from the *T*. *vaginalis* LDT; significant differences between each group compared to the pre-freeze CP values are denoted with an asterisk (ANOVA with Dunnett’s Multiple Comparison Test; p<0.05).

We examined the characteristics of DNA extracts of ThinPrep PreservCyt specimens from spin columns and the automated cobas x 480 instrument processed in parallel. With standard spectrophotometry, we observed that DNA concentrations were significantly lower in spin column-based preparations (126.2 vs 11.93 ng/μL, p<0.05; Fig 3A), but were characterized by significantly increased A_260_/A_280_ ratios (1.02 vs 1.75, p<0.05; Fig 3B). To address DNA eluate stability, once-weekly freeze-thaw cycles of spiked x 480-purified DNA specimens (up to 21 days with or without magnetic glass particles) resulted in a modest (approximately 1 cycle) increase in CP values compared to the pre-freeze values (ANOVA with Dunnett’s Multiple Comparison Test, p<0.05; Fig 4). Similar stability results were observed for DNA purified using spin columns (data not shown). A panel of LDT-positive/negative samples (n = 20 each; ascertained using residual x 480 eluates) was then extracted using spin columns in order to assess whether LDT results differed between the extraction methods, with the goal of validating both extraction methods for diagnostic use in the future. Perfect agreement of LDT results was observed between the extraction methods (Kappa = 1.00), and so therefore the comparative assessment to the TIB MOLBIOL NAATs was performed using residual DNA eluates from the automated x 480 instrument.

### Diagnostic utility and *T*. *vaginalis* organism burden

ThinPrep PreservCyt specimens and residual DNA eluates (n = 200) were obtained from University Medical Center in New Orleans, LA. The median age among subjects in this study was 45 years; we were unable to stratify *T*. *vaginalis* results by age because specimens were de-identified prior to testing. Prior to *T*. *vaginalis* PCR, a separate PCR assessment of human β-globin was used to assess specimen adequacy; β-globin was not detected in 1 specimen (0.5%), which was excluded from subsequent analyses. Using residual x 480 DNA eluates, the prevalence of *T*. *vaginalis* was 7.0% in this study of Louisiana women (14/199). The mean *T*. *vaginalis* organism burden was 1.0x10^6^ ± 4.5x10^5^ organisms per mL of ThinPrep PreservCyt. *T*. *vaginalis* testing was performed after T2000 cytology processing, and no pre/post-cytology comparison was performed in this study. As such, it is unknown whether cytology processing impacted the accuracy of the *T*. *vaginalis* organism burden calculations.

### Comparative assessment of the *T*. *vaginalis* LDT and Two TIB MOLBIOL NAATs

In order to assess diagnostic utility, the *T*. *vaginalis* LDT was compared directly to two commercially available qualitative NAATs from TIB MOLBIOL: the LightMix *Trichomonas vaginalis* test; and (2) LightMix Modular Dx *Trichomonas vaginalis* Repeat DNA test. Residual DNA eluates from the x 480 instrument were used for the *T*. *vaginalis* LDT and, per the package insert, spin column DNA preparations were used for both TIB MOLBIOL tests. The *T*. *vaginalis* LDT was compared independently to each of the TIB MOLBIOL tests. Test results did not differ between the TIB MOLBIOL tests. Sensitivity, specificity, PPV, and NPV of the LDT versus each TIB MOLBIOL test as independent reference methods (gold standard) were all >92% (Table 2). The LDT failed to detect *T*. *vaginalis* DNA in a single specimen that was positive by both TIB MOLBIOL tests, and was confirmed to be *T*. *vaginalis*-positive by follow up Aptima testing. Re-testing this discrepant specimen with the LDT produced the same negative result. A single specimen produced a positive result with the LDT, but was negative by both TIB MOLBIOL tests. Aptima testing confirmed this LDT-positive/TIB MOLBIOL-negative specimen to be negative for *T*. *vaginalis*, which was classified as a false-positive LDT result. Due to the study design of using Aptima to resolve discordant results, all Aptima testing was performed between 45 and 60 days after specimen collection, which is outside of the 30-day indication in the package insert. Collectively, discrepant analysis using Aptima confirmed the initial results and therefore did not alter the results presented in Table 2.

**Table 2:** Comparative evaluation of the *T*. *vaginalis* LDT versus two TIB MOLBIOL NAATs as reference methods**.

	LightMix *Trichomonas vaginalis*	LightMix Modular Dx *Trichomonas vaginalis* Repeat DNA
LDT Results, % Sensitivity	13/14, 92.9 (0.641–0.996)	13/14, 92.9 (0.641–0.996)
LDT Results, % Specificity	184/185, 99.5 (0.966–1.000)	184/185, 99.5 (0.966–1.000)
% NPV* (95% CI)	99.5 (0.965–1.000)	99.5 (0.965–1.000)
% PPV* (95% CI)	92.9 (0.641–0.996)	92.9 (0.641–0.996)
Kappa (95% CI)	0.923 (0.818–1.000)	0.923 (0.818–1.000)

* NPV, negative predictive value; PPV, positive predictive value.

** LDT results were compared independently to each of the TIB MOLBIOL NAATs, which served as the gold standard comparator for these calculations.

As a secondary outcome, we compared the LDT to cytological detection of *T*. *vaginalis* and observed sensitivity and specificity values of 57.1 and 100.0%, respectively (Kappa = 0.713, [95% CI = 0.496–0.930]). Six specimens were deemed positive by the LDT but negative during the cytology screen, each of which also tested positive with both TIB MOLBIOL tests and Aptima.

## Discussion

We observed a prevalence of 7.0% in this generally low-risk population of Louisiana women. Among these women, the *T*. *vaginalis* burden in the cervix was extraordinarily high, with a total mean parasite load of more than 1x10^7^ organisms per specimen. All current FDA-approved *T*. *vaginalis* tests generate qualitative results, and despite having the capacity to ascertain organism burden with this assay system, we use the LDT qualitatively in our clinical diagnostic laboratory. The quantitative capacity of the test is primarily applicable for clinical research as we continue to investigate the pathobiology of *T*. *vaginalis* infections. With the option of spin column or automated extraction methods, and rapid one-hour PCR run time, the LDT dovetailed nicely with our regular STI diagnostics workflow. The use of otherwise-discarded DNA eluates from cobas HPV testing was cost-saving as this workflow eliminated the need for a separate DNA extraction for *T*. *vaginalis* testing, while spin-columns met our need to process specimens not initially submitted for HPV testing. Our rationale for comparing DNA quantity and purity between spin columns and x 480 eluates was to garner a basic understanding of whether spin-column DNA differs from the automated magnetic glass particle-based method. Ultimately, residual DNA eluates were used for LDT testing and compared to TIB MOLBIOL results generated using spin column-purified DNA (i.e. the extraction method differed). This approach was used for three reasons: (1) it ensured that spin columns were used for the TIB MOLBIOL NAATs as indicated in the package insert; (2) using residual DNA eluates remaining after HPV testing was more cost-effective than re-purifying the specimens using spin columns for study purposes; and (3) our sub-study herein showed that LDT results did not differ between DNA purification methods.

Direct comparison of the LDT with the TIB MOLBIOL NAATs showed generally good performance with all measures of concordance > 92%. The small sample size for this study, despite the 7% prevalence, resulted in relatively few *T*. *vaginalis*-positive specimens (n = 14). In turn, the single LDT false-positive result reduced the sensitivity to 92% suggesting that validating laboratories may consider expanding the validation sample size, particularly if serving populations with low *T*. *vaginalis* prevalence. Overall, only two specimens produced discordant results in this study (1.01%), which we then adjudicated using the Aptima *T*. *vaginalis* test. The Aptima test targets highly abundant rRNA molecules, and therefore theoretically should have superior analytical sensitivity compared to our single-copy target LDT. With Aptima testing, one of the discrepant specimens was deemed a LDT false-positive, and the other a false-negative. Although the Aptima test deemed the single LDT-positive/TIB MOLBIOL-negative specimen a false-positive, Aptima testing was performed between 45–60 days after specimen collection, which is greater than the 30 day maximum noted in the package insert. Therefore, it cannot be ruled out that the Aptima test would have produced a positive result if performed as intended. It is also possible that the LDT false-positive result was due to cross-reactivity to *T*. *tenax*, which was not identified during *in silico* analyses since NCBI sequence data was unavailable. However this normal inhabitant of the oral cavity is very rarely detected in the lower urogenital tract [18, 19]. The single TIB MOLBIOL-positive/LDT-negative specimen was confirmed to be *T*. *vaginalis*-positive by Aptima. Considering analytical sensitivity, the TIB MOLBIOL tests target a locus repeated eight times in the genome, and therefore, it is likely that both the Aptima and TIB MOLBIOL tests have superior analytical sensitivity compared to our LDT. This apparent inferiority of the LDT may in part be attributed to the single-copy genomic target, and using only 5 μL of the relatively large elution volume (150 μL) from the cobas HPV test. Laboratories using this LDT may elect to perform a method comparison study with an FDA-approved test and/or further optimize the LDT reaction parameters based on screening versus diagnostic utility, population prevalence, and desired analytical sensitivity.

Overall, the LDT workflow was time-efficient and the manual reaction setup was acceptable to laboratory staff. Similar to other LDTs, an inherent level of technical expertise and comfort with molecular biology are required to execute such tests. After establishing run templates and macros within the software, technicians within our laboratory found the UDF software to be streamlined and efficient for routine testing. An internal control that is co-amplified in the same reaction well would be more cost-effective than the detection of human β -globin on a separate run as in this study; developing this duplex assay is a current priority in our laboratory.

Not surprisingly, this study confirms the enhanced sensitivity of NAATs compared to direct microscopic diagnosis of *T*. *vaginalis* (57.1% versus the LDT)[12]. Papanicolaou smears are performed chiefly to detect signs of cervical cancer or pre-cancerous lesions, and direct the need for follow-up colposcopy and biopsy of the cervix. However, the presence/absence of *T*. *vaginalis* is a required assessment criterion in our hospital and this result is included on each cytology report. We observed cytological detection to be highly specific for diagnosis (100% versus the LDT), however identification of *T*. *vaginalis* on Pap smears is not a recommended screening method. Additionally, although direct microscopic *T*. *vaginalis* result results are included on the cytology report to the requesting physician, re-screening by wet mount or validated NAAT should ultimately direct therapy. In order to reduce experimenter bias during cytology, *T*. *vaginalis* results from Papanicolaou microscopy were retrospectively obtained from cytology reports. Therefore, upon initial screening the cytologists were unaware the specimen was part of a research study. Subsequently, each LDT-positive/cytology-negative specimen was re-analyzed in a blinded manner by staff cytologists, and despite disclosing that the re-evaluation was to clarify *T*. *vaginalis* results, no results differed from the initial screening. Taken together, we are confident this comparative assessment to cytology-based detection is accurate and representative of routine laboratory testing.

Collectively, this study highlights our LDT as an accurate, user-friendly, and reliable method for qualitative *T*. *vaginalis* detection in ThinPrep PreservCyt specimens. To our knowledge, this is the first report of residual DNA eluates from cobas HPV testing being tasked for *T*. *vaginalis* detection, and proved to be a streamlined solution for reflex testing. Importantly, this study provides important evidence for the significant *T*. *vaginalis* organism burden of the cervix, which begs the question of whether organism burden relates to clinical signs and severity of lower genital tract disease. The capacity to quantify parasite load is a valuable tool in the research setting. Collectively, although we await final guidance from the FDA on the future oversight of LDTs for clinical diagnosis, the LDT described herein will, for the foreseeable future, be useful for the continued investigation of *T*. *vaginalis* infections on reproductive health, adverse pregnancy outcomes, and the mechanisms of enhanced HIV transmission [20, 21].

## Supporting information

S1 Dataset Dataset for Figs 1–4. (XLSX)
